# Obesity is associated with distinct oxylipins in heart failure with preserved ejection fraction

**DOI:** 10.1007/s11306-026-02449-x

**Published:** 2026-05-11

**Authors:** Ashish Yadav, Sareeta Manandhar, Alex Kloster, Vaishnavi Aradhyula, Krishna Rao Maddipati, George V. Moukarbel, David J. Kennedy, Samer J. Khouri, Rajesh Gupta

**Affiliations:** 1 Division of Cardiovascular Medicine, University of Toledo, Toledo, OH USA; 2 Department of Internal Medicine, Cleveland Clinic, Cleveland, OH USA; 3 Department of Pathology, Lipidomic Core Facility, Wayne State University, Detroit, MI USA

**Keywords:** Heart failure with preserved ejection fraction, Obesity, Oxylipins, Pulmonary hypertension, Diols

## Abstract

**Background:**

Heart failure with preserved ejection fraction (HFpEF) accounts for nearly half of heart failure cases, with obesity emerging as a key pathophysiologic driver. Oxylipins are lipid signaling molecules linked to adverse HFpEF outcomes, but their relationship with obesity remains unclear.

**Objectives:**

To evaluate association between obesity and arterialized and venous oxylipins in HFpEF patients.

**Methods:**

In this prospective single-center cohort study, 90 patients with HFpEF underwent transthoracic echocardiography and right heart catheterization with arterialized pulmonary capillary and venous blood sampling. Serum oxylipins were quantified via liquid chromatography-mass spectrometry. Oxylipin levels were log-transformed and standardized. Oxylipin associations with obesity (BMI ≥ 30 kg/m²) were evaluated using logistic regression, and pulmonary hypertension (PH) subgroup analyses using a one-sample proportion test.

**Results:**

Of the 90 patients, 61 (67.8%) were obese. Obese patients were younger, more frequently female and African American, had higher prevalence of diabetes (54.1% vs. 17.2%, *p* < 0.001) and exhibited greater interventricular septal and posterior wall thickness. Multivariable and volcano plot analyses demonstrated inverse associations between obesity and several arterialized oxylipins, including 12,13-DiHOME (OR:0.48; *p* = 0.03), 19,20-DiHDoPE (OR:0.53; *p* = 0.03), 11,12-DiHETrE (OR:0.31, *p* = 0.04), 9,10-DiHOME (OR;0.42; *p* = 0.02), and 5(S),6(S)-DiHETE (OR:0.51; *p* = 0.02). In contrast, arterialized 19R-hydroxy-prostaglandin E1 was positively associated with obesity (OR:1.91, *p* = 0.03). Among venous oxylipins, 15(R)-PGE1 (OR:2.23; *p* = 0.02) and 19R-hydroxy-prostaglandin E1 (OR:1.91; *p* = 0.02) showed positive associations with obesity. Obese patients constituted a higher proportion of combined pre- and post-capillary PH subgroup (*p* = 0.003).

**Conclusions:**

Obesity in HFpEF is associated with reduced oxylipin diol levels, higher levels of prostaglandin E1 metabolites, and higher burden of pulmonary hypertension.

**Graphical abstract:**

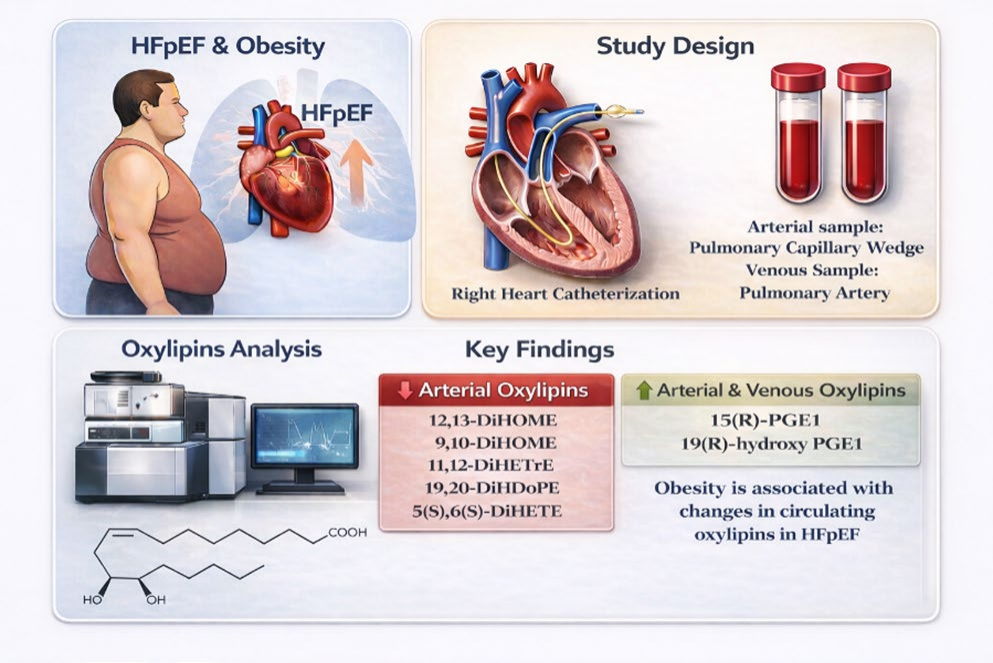

## Introduction

Heart failure with preserved ejection fraction (HFpEF) accounts for almost half of all heart failure cases and is driven largely by aging populations and increasing burden of obesity and associated metabolic diseases (Owan et al., 2006, Dunlay et al., 2017). HFpEF involves complex interactions among cardiac, vascular, renal and metabolic systems (Shah et al. 2015).

Obesity is highly prevalent in HFpEF and is increasingly recognized as a driver rather than just a coincidental comorbidity (Packer et al., 2020). Adipose tissue functions as an active endocrine organ, releasing adipokines, inflammatory cytokines, and lipid signaling molecules. These promote systemic inflammation, myocardial fibrosis, endothelial dysfunction and impaired ventricular relaxation (Paulus & Tschöpe, 2026). Obesity is also associated with renal sodium retention, plasma volume expansion, and increased cardiac filling pressures (Pandey et al. 2018). Obesity related pulmonary vascular remodeling contributes to pulmonary hypertension and right ventricular (RV) dysfunction which are the key determinants of exercise intolerance and adverse outcomes in HFpEF (Obokata et al., 2017).

Oxylipins are lipid signaling molecules derived from polyunsaturated fatty acids (PUFA) that regulate inflammation, vascular tone, and metabolic pathways (Gabbs et al., 2015). Recent studies have demonstrated that circulating oxylipins predict mortality in HFpEF and are associated with impaired RV–pulmonary artery coupling, thus highlighting their relevance for HFpEF pathogenesis and outcomes (Aradhyula et al., 2025, Kloster et al., 2025). However, the relationship between obesity and circulating oxylipins in HFpEF remains poorly defined. Therefore, we sought to assess oxylipin profiles in obese and non-obese participants with HFpEF using both arterialized pulmonary capillary blood and mixed venous samples. This dual-sampling approach across the pulmonary circulation allowed us to identify localized trans-pulmonary differences.

## Methods

This study was designed as a prospective observational study at the University of Toledo Medical Center. The Institutional Review Board of University of Toledo approved both the study methods and research protocol. Each patient provided written informed consent before enrolling. All procedures adhered strictly to ethical and institutional guidelines.

### Study population and enrollment

This single center study enrolled 90 patients over a span of 8 months ranging from August 1, 2011 until March 30, 2012. Eligible participants were adults aged 18 and above with a new clinical diagnosis of HFpEF. HFpEF was defined by the presence of heart-failure signs and symptoms along with an echocardiographic LVEF ≥ 50%. Patients only in NYHA class II or III and without a prior history of malignancy were considered for participation. Patients were excluded if they had either HFrEF or LVEF < 50%, end-stage renal disease on hemodialysis, a history of myocardial infarction, severe liver disease or prior cancer. Elevated BNP levels were not required for the diagnosis, as normal values do not preclude HFpEF. Right heart catheterization was performed based on standard clinical indications, as determined independently by the patient’s treating clinical cardiologist. Our study cohort consisted exclusively of eligible patients with HFpEF who were already scheduled to undergo RHC for clinical reasons. These patients were subsequently approached and provided written informed consent prior to study enrollment and blood sampling. Although pulmonary capillary wedge pressure (PCWP) was measured in all participants, an elevated PCWP was not used as an inclusion criterion to allow enrollment of HFpEF patients who were compensated at the time of the right heart catheterization procedure. Obesity was defined as body mass index (BMI) ≥ 30 kg/m^2^.

### Data collection, procedures, and follow-up

All participants underwent baseline right heart catheterization (RHC) and transthoracic echocardiography. Demographic characteristics, cardiovascular risk factors and comorbidities were obtained from the electronic health record through independent chart review by two study team members.

### RHC mixed venous and arterialized pulmonary capillary blood sampling

Following standard overnight fast prior to the procedure, all enrolled patients underwent RHC and a comprehensive echocardiographic evaluation. During RHC, blood samples were obtained via the pulmonary artery catheter. The catheter was advanced to the pulmonary capillary wedge position with the balloon inflated. Correct positioning was confirmed by the presence of a pulmonary capillary wedge pressure (PCWP) waveform. Next, to further confirm the wedge position, 10 mL of blood was discarded, and oxygen saturation was measured to ensure a value > 90%. Once this was confirmed, then approximately 19 mL of blood was collected from the distal port of the pulmonary artery catheter while in the PCW position. This sample, representing left atrial blood, was designated as the arterialized pulmonary capillary blood sample. The balloon was then deflated and the catheter was withdrawn until a pulmonary artery waveform was observed. Again, 10 mL of blood was discarded, followed by measurement of oxygen saturation. Mixed venous blood was then collected from the distal port, with approximately 19 mL obtained. This sample was labeled as the venous sample.

All samples were immediately placed on ice and transported to the laboratory within 1 h for processing. Serum was separated by centrifugation, placed into 2 mL tubes, flash-frozen in liquid nitrogen and stored at − 80 °C until analysis. Plasma samples were preserved for future studies but were not utilized in the present analysis.

### Serum processing and lipidomic assessment

Serum obtained from both arterialized pulmonary capillary blood and mixed venous sampling was analyzed at the Lipidomics Core facility at Wayne State University using the methods published by Maddipati et al. (Kloster et al. 2025). Briefly, samples were adjusted to a final volume of 1 to 2 mL and spiked with a mixture of internal standards including 15(S) HETE d8, leukotriene B4 d4, resolvin D2 d5, 14(15) EpETrE d11 and prostaglandin E1 d4 at 5 ng each to enable recovery assessment and quantitative analysis. Samples were mixed thoroughly and polyunsaturated fatty acid metabolites were extracted using C18 solid phase extraction columns as described. High performance liquid chromatography was performed using a Luna C18 column (3 µ, 2 × 150 mm) and the eluate was directly introduced into the electrospray ionization source of a QTRAP 5500 mass spectrometer operated in negative ion mode. Data acquisition and quantification of multiple reaction monitoring transition chromatograms were carried out using MultiQuant software version 3.3. For each detected lipid mediator, mass spectra were acquired using the Enhanced Product Ion mode to confirm analyte identity based on spectral features in addition to matching multiple reaction monitoring transitions and retention times with the standard. Internal standard signals were used to normalize extraction efficiency and to enable relative quantification of individual analytes.

### Echocardiography

Echocardiographic measurements were performed according to the recommendations of the American Society of Echocardiography using Philips IE33 and GE Vivid 7 systems and all images were interpreted on the EchoPAC workstation. At baseline, we also documented the ejection fraction, left atrial volume index, intraventricular septal thickness, posterior wall thickness, tricuspid annular plane systolic excursion, right ventricular systolic pressure and the E/e’ prime ratio as an indicator of left ventricular filling pressures.

### Statistical analysis

Continuous variables were summarized as median with interquartile range and categorical variables were reported as counts and percentages. Comparisons between the two groups, non-obese (BMI < 30 kg/m^2^) and obese (BMI ≥ 30 kg/m^2^), were made using the Wilcoxon rank-sum test for continuous variables and Fisher’s exact test for categorical variables.

Oxylipin concentrations were natural log-transformed and then standardized to a mean of 0 and a standard deviation of 1, to address right-skewness and account for differences in scale. The volcano plots were then generated adjusting oxylipins for age, sex, race and diabetes to visualize oxylipins associated with obesity. Odds ratios (ORs), along with corresponding 95% confidence intervals (CIs) and *p*-values, were estimated for each oxylipin using binary logistic regression models.

Oxylipins that differed significantly between non-obese and obese groups (*p* < 0.05), as well as those identified as significant in unadjusted and adjusted volcano plot analyses, were included in subsequent multivariable regression analyses. Separate adjusted logistic regression models were constructed for each oxylipin, with obesity status (BMI ≥ 30 vs. < 30 kg/m^2^) specified as the binary outcome. Covariates included in each model were selected based on statistical significance in univariable analyses (*p* < 0.05) as well as their clinical relevance. Oxylipin concentrations were natural log-transformed and standardized to a mean of 0 and standard deviation of 1 prior to modelling.

A pie chart was generated to illustrate the distribution of obesity status among participants with combined post-capillary pulmonary hypertension (CpcPH). To evaluate whether the proportion of obese individuals differed from an expected value of 50%, a one-sample proportion test was performed. All statistical tests were two-sided, and a *p*-value < 0.05 was considered statistically significant. All analyses were performed using R version 4.5.1.

For multivariable regression models, patients with missing covariate data were excluded from the analysis. Specifically, one participant was missing data for the race covariate and was thus excluded from the adjusted models, resulting in a sample size of 89 for that specific analysis.

## Results

### Baseline characteristics

Of the 90 patients in our HFpEF cohort, 61 (67.8%) were obese (BMI ≥ 30 kg/m²). Table 1 compares the baseline characteristics between non-obese patients (*n* = 29) and obese patients (*n* = 61). The obese group had a higher percentage of females (77% vs. 51.7%) and was significantly younger, with a median age of 66 years compared with median age of 76 years in the non-obese group. The obese group had significantly more African American patients than the non-obese group (33.3% vs. 6.9%) and a higher prevalence of diabetes (54.1% vs. 17.2%). There were no significant differences in the prevalence of coronary artery disease, hypertension, hyperlipidemia or CKD. Both the groups had similar blood pressure and heart rate. Laboratory findings revealed that obese group had significantly higher platelet count (233 × 10³/µL vs. 199 × 10³/µL) and significantly lower BNP levels (115 pg/ml vs. 366.5 pg/ml). Other lab values including hemoglobin levels, sodium levels, potassium levels, creatinine, blood urea nitrogen and GFR were not statistically different. There were no statistically significant differences for medication usage including beta-blockers, ACE inhibitors, angiotensin receptor blockers and phosphodiesterase inhibitors. However, diuretic use was more common in the obese group (65.6% vs. 34.5%).

**Table 1:**
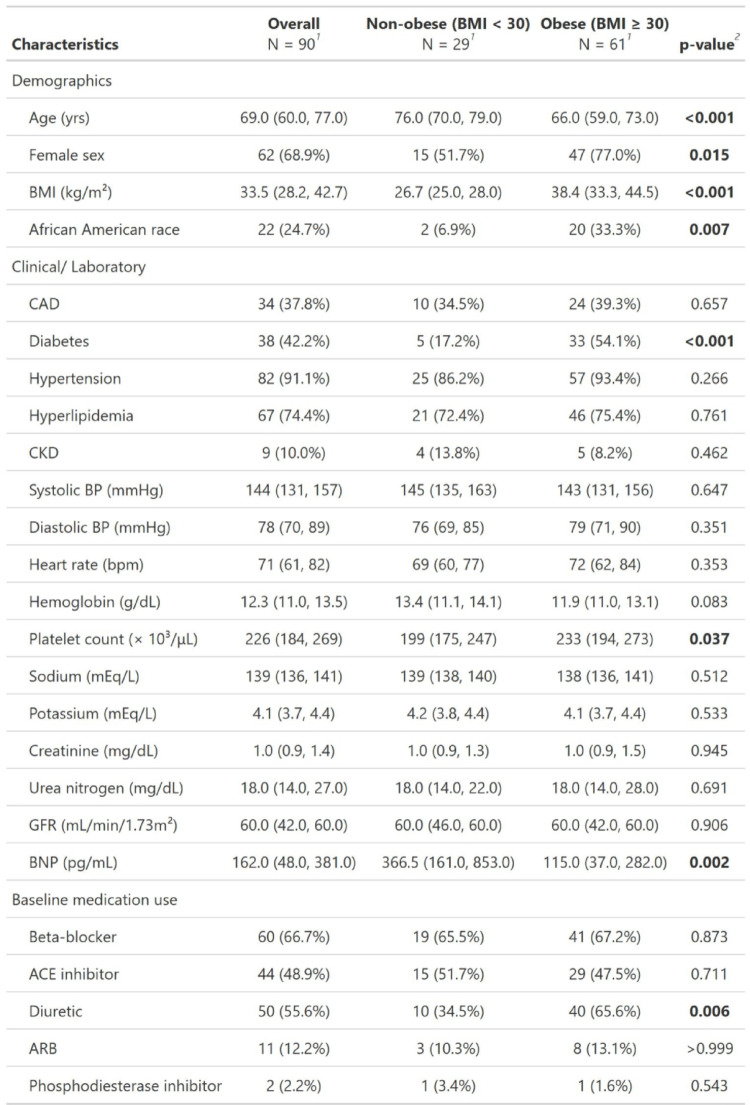
Baseline characteristics

^1^Median (Q1, Q3); n (%)

^2^Wilcoxon rank sum test; Pearson's Chi-squared test; Fisher's exact test

Baseline demographic, lab values and medication usage of heart failure with preserved ejection fraction (HFpEF) patients grouped according to BMI status

Values are displayed as median [25th percentile–75th percentile]

*BMI* body mass index, *CAD* coronary artery disease, *CKD* chronic kidney disease, *BP* blood pressure, *ACE inhibitor* angiotensin converting enzyme inhibitor, *ARB* angiotensin II receptor blocker

### Baseline RHC and echocardiographic parameters of obesity in HFpEF

Table 2 compares RHC and echocardiographic parameters between the obese and non-obese groups. There was a statistically significant difference in mean right atrial pressure between the obese and non-obese groups (*p* = 0.045). Right ventricular systolic pressure, mean pulmonary artery pressure and pulmonary artery wedge pressure were numerically higher in the obese group, though these differences were not statistically significant. On echocardiogram, we found larger interventricular septal diameter (1.2 cm vs. 1 cm, *p* = 0.015) and larger posterior wall diameter in the obese group (1.1 cm vs. 0.9 cm, *p* < 0.001). Other echocardiographic parameters such as ejection fraction, left atrial volume index, tricuspid annular plane systolic excursion and E/e′ ratio didn’t differ significantly between the groups.

**Table 2:**
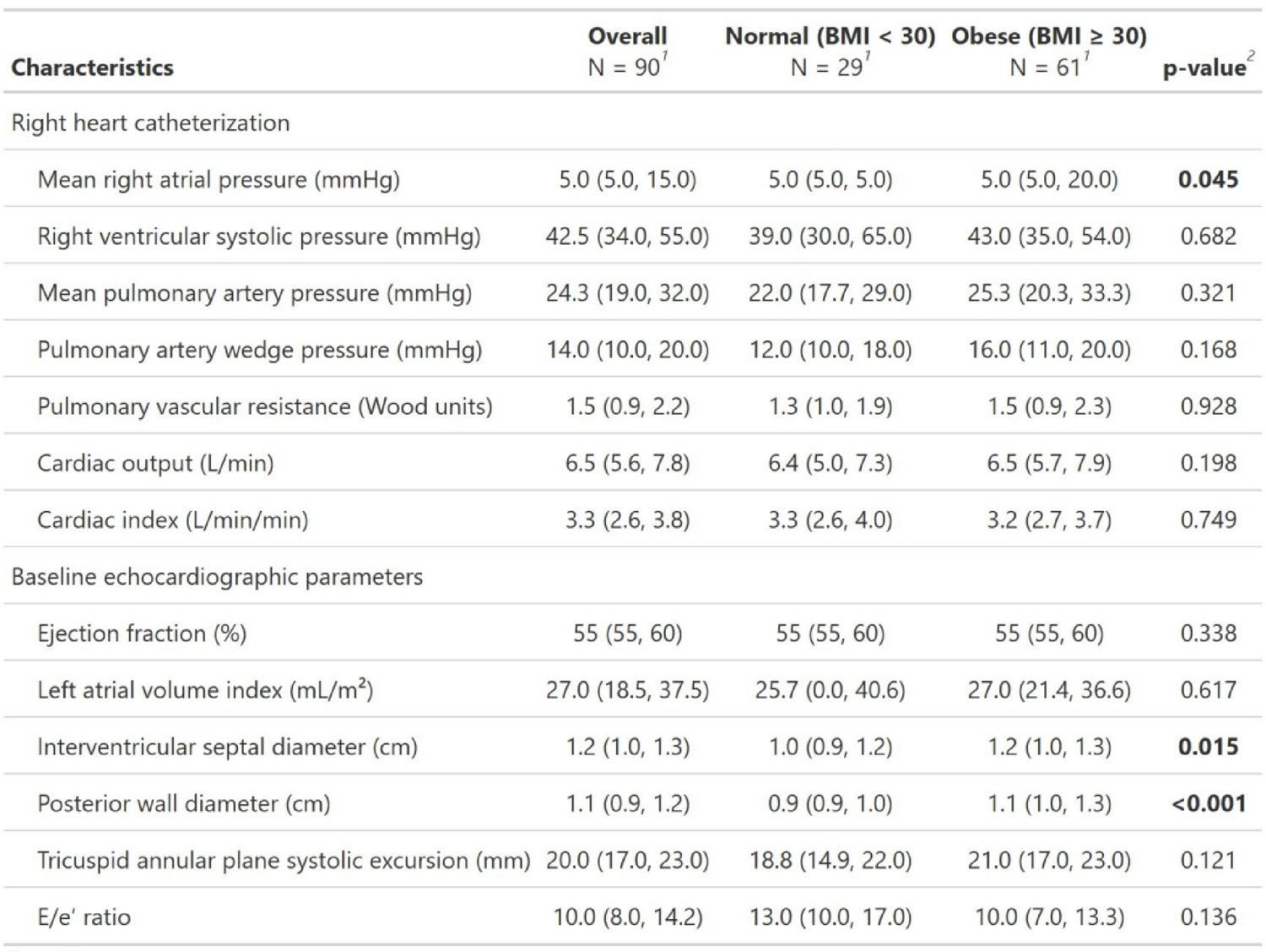
Right heart catheterization and echo parameters

^1^Median (Q1, Q3)

^2^Wilcoxon rank sum test

Baseline right heart catheterization and echocardiographic parameters of non-obese and obese patients

### Oxylipins and obesity

**Fig. 1:**
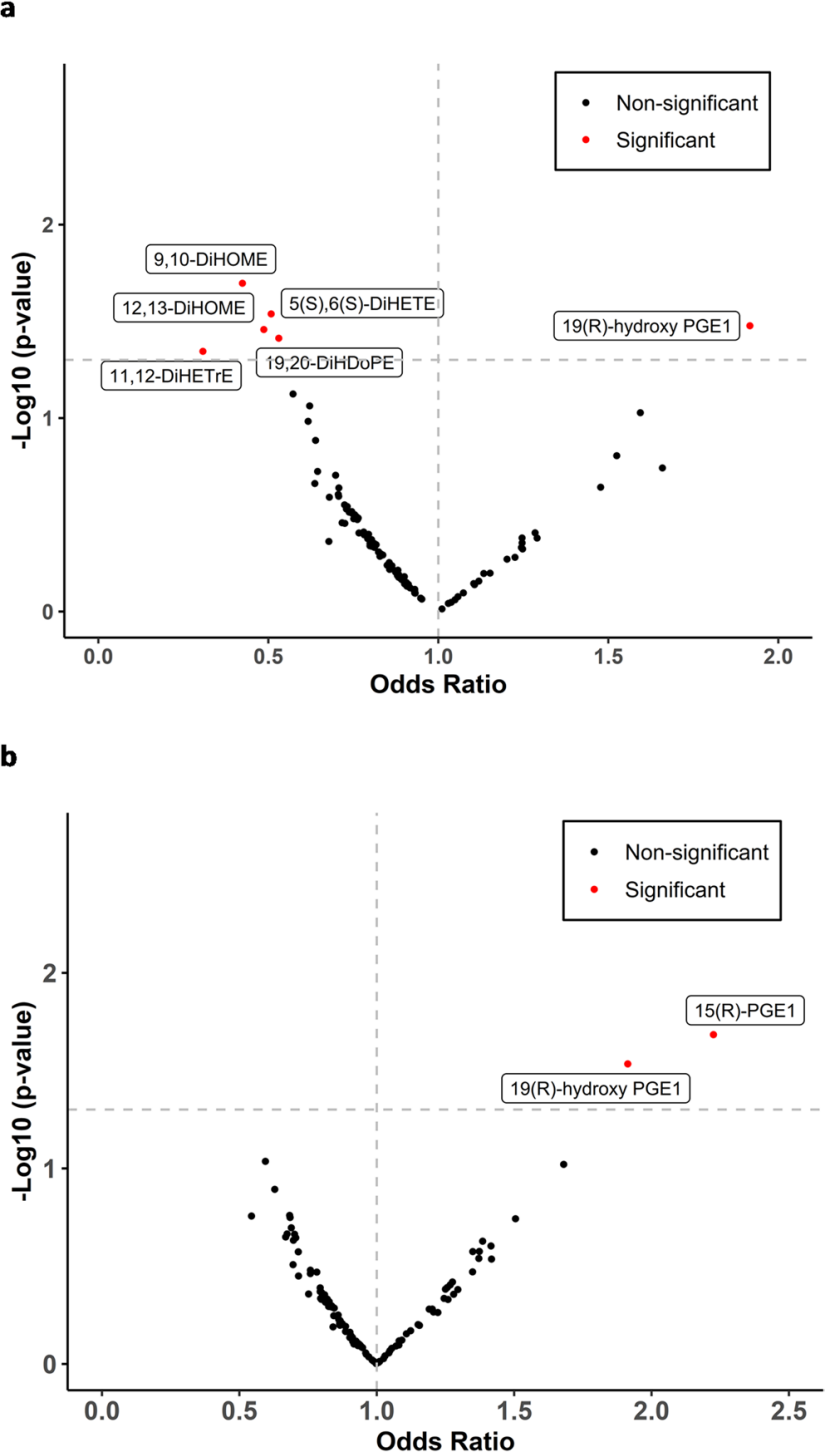
Volcano plot visualization of 143 Arterialized Pulmonary Capillary blood **a** and Mixed Venous blood **b** oxylipins assessing the relationship between oxylipins and obesity after diagnosis of heart failure with preserved ejection fraction (HFpEF), adjusted for age, sex, race and diabetes

Volcano plot analysis of arterialized pulmonary capillary blood oxylipins revealed several oxylipins that were significantly associated with obesity (Fig. 1a and b). The following oxylipins demonstrated a negative correlation with obesity: 12,13-DiHOME (OR 0.48, 95% CI 0.24–0.92; *p* = 0.03), 19,20-DiHDoPE (OR 0.53, 95% CI 0.28–0.95; *p* = 0.03), 11,12-DiHETrE (OR 0.31, 95% CI 0.09–0.78; *p* = 0.04), 9,10-DiHOME (OR 0.42, 95% CI 0.19–0.84; *p* = 0.02) and 5(S),6(S)-DiHETE (OR 0.51, 95% CI 0.27–0.91; *p* = 0.02). In contrast, arterial 19R-hydroxy-prostaglandin E1 (OR 1.91, 95% CI 1.07–3.61; *p* = 0.03) and venous oxylipins 15(R)-PGE1 (OR 2.23, 95% CI 1.17–4.66; *p* = 0.02) and 19R-hydroxy-prostaglandin E1 (OR 1.91, 95% CI 1.09–3.53; *p* = 0.02) were positively correlated with obesity. In a multivariable model adjusted for age, sex, race and diabetes, specific-oxylipins, including 12,13-DiHOME, remained independently associated with obesity (Table 3).

**Table 3:**
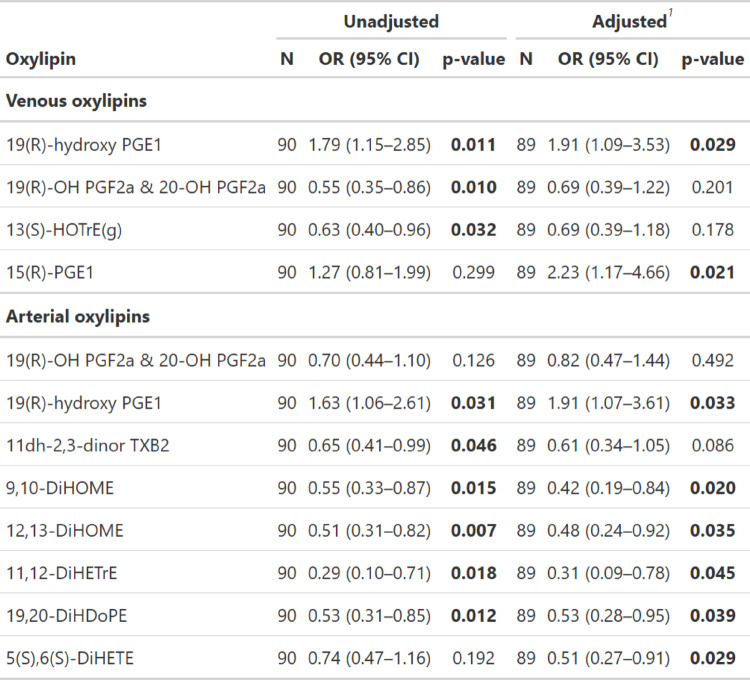
Association between arterialized pulmonary capillary and mixed venous oxylipins with Obesity

^1^Model adjusted for age, sex, race and diabetes

*CI* confidience interval, *OR* odds ratio

### Obesity and pulmonary hypertension

**Fig. 2:**
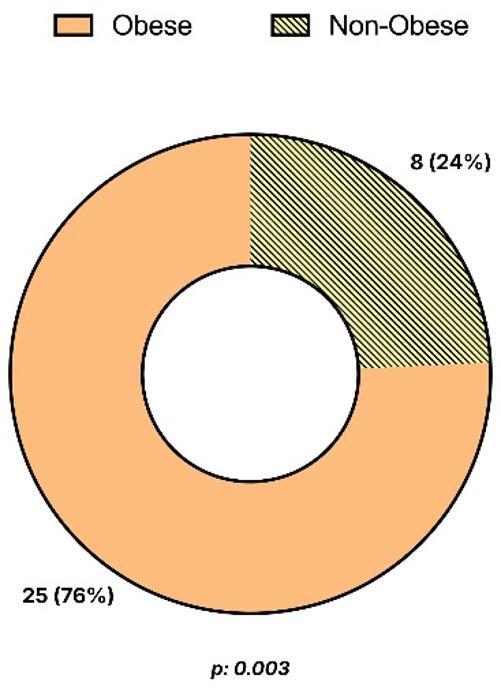
Proportion of obese vs. non-obese participants in HFpEF with combined pre- and post-capillary pulmonary hypertension (CpcPH)

The distribution of combined pre- and post-capillary pulmonary hypertension (CpcPH) differed by obesity status, as shown in Fig. 2. Patients with obesity constitute a significantly higher proportion of CpcPH compared with non-obese patients (76% vs. 24%; *p* = 0.003).

## Discussion

In this prospective cohort study of patients with HFpEF, we identified novel oxylipins that are associated with obesity. Specifically, lower arterialized pulmonary capillary levels of several oxylipin diols including 12,13-DiHOME and higher levels of prostaglandin E1 metabolites in both arterialized pulmonary capillary and mixed venous blood were significantly associated with obesity in HFpEF patients.

Obesity has emerged as one of the most important and prevalent contributors to the development and progression of HFpEF. Epidemiologic data indicates that more than half of patients hospitalized with HFpEF are overweight or obese and obesity independently increases the risk of incidence of heart failure even after adjustment for hypertension, diabetes and coronary artery disease (Shah et al., 2015, Maddipati et al., 2014, Kenchaiah et al., 2002). Although HFpEF is identified as a heterogeneous clinical syndrome, obesity-related HFpEF represents a dominant subtype (Shah et al., 2015, Obokata et al., 2017).

Several studies have further shown that obesity is associated with increased HFpEF hospitalization rates and worse symptom burden. Excess adiposity promotes chronic low-grade inflammation through adipokine dysregulation, immune cell activation, and oxidative stress. This leads to multiple adverse changes including coronary microvascular dysfunction, impaired nitric oxide signaling, and myocardial fibrosis, which are key features of HFpEF pathophysiology (Franssen et al., 2016, Mohammed et al., 2015). Recent studies have emphasized that HFpEF should be viewed as a systemic, cardiometabolic disorder rather than a purely cardiac disease, with obesity acting as a central driver (Shah et al., 2015, Packer et al., 2020).

Increasing attention is directed toward metabolic intermediates and lipid-derived signaling molecules as potential mediators of obesity related myocardial dysfunction. Alterations in lipid signaling have been implicated in HFpEF development (Neubauer, 2007). Oxylipins represent an underexplored link between obesity, inflammation and HFpEF. This study adds to this growing literature by demonstrating differences in arterialized pulmonary capillary and mixed venous oxylipin profiles among obese and non-obese patients with established HFpEF. These findings further reinforce the concept that obesity is not merely a comorbidity, but instead a fundamental modifier of HFpEF biology.

Oxylipins are lipid mediators derived from polyunsaturated fatty acids (PUFAs) and metabolized through the cytochrome P450 (CYP), cyclooxygenase (COX) and lipoxygenase (LOX) pathways. They are essential in regulating vascular tone, inflammation, and cardiometabolic homeostasis (Gabbs et al., 2015, Nayeem, 2018). Within the cytochrome P450 pathway, epoxides such as epoxyeicosatrienoic acids (EETs) are converted to their corresponding diols via hydrolysis by soluble epoxide hydrolase (sEH) (Spector & Kim, 2015). In our study, several arterialized pulmonary capillary diols, including 12,13-DiHOME, were significantly lower in obese HFpEF patients compared with non-obese patients. This suggests a systematic alteration in epoxide–diol balance associated with obesity in HFpEF. This may also represent alterations in sEH activity or could represent uptake of diols within the pulmonary circulation since the lower levels of diols were found in the wedge/left atrial (post-lung) blood samples.

Prior studies of other diseases have reported reduced circulating oxylipin diol concentrations in obesity and metabolic syndrome. This reflects potential alteration of sEH activity or adaptive metabolic reprogramming (Hateley et al., 2024, Vasan et al., 2019, Gurup et al., 2023). Diols such as 12,13-DiHOME have been implicated in fatty acid uptake and mitochondrial metabolism, with context-dependent effects across tissues (Stanford et al., 2018, Lynes et al., 2017). It remains unclear whether reduced diol levels represent a loss of protective signaling or an adaptive response for preserving upstream epoxides.

The role of sEH in HFpEF remains incompletely understood. Most of the existing evidence comes from preclinical studies, where sEH inhibition has been associated with improvements in endothelial function and modest improvements in diastolic relaxation (Zhang et al., 2026, Peng et al., 2022). Direct human data linking sEH activity to HFpEF is sparse. Hence, our observation of lower circulating diol levels in obese HFpEF patients should be interpreted cautiously. Rather than implying a direct pathogenic role, these findings reflect a broader alteration in lipid metabolism associated with obesity. Studies which involve direct assessments of sEH activity, oxylipin uptake or production in the pulmonary circulation, myocardial function, and clinical outcomes will be necessary to understand the clinical significance of altered oxylipin diol levels in obese HFpEF patients.

Besides the diols, 19(R)-hydroxy-PGE1 showed a consistent positive association with obesity in HFpEF across both arterialized pulmonary capillary and mixed venous samples. This suggests a systemic shift toward prostaglandin pathway activation in obese HFpEF. Prostaglandin E1 related metabolites also exert important vasoregulatory and cytoprotective effects that may be relevant in advanced cardiometabolic disease. Experimental and clinical studies have shown that PGE1 signaling promotes vasodilation, improves endothelial function, inhibits platelet aggregation and attenuates ischemia-related injury, thus supporting a protective role at the vascular level (Makita et al., 1997, Kreutz et al., 2013, Fang et al., 2010). Consistent with these effects, prior analysis have demonstrated that higher circulating levels of 19(R)-hydroxy-PGE1 are associated with reduced mortality risk. In this context, the observed elevation of 19(R)-hydroxy-PGE1 in obese HFpEF patients may represent an adaptive response to heightened inflammatory and hemodynamic stress, thus, potentially mitigating downstream vascular injury and adverse clinical outcomes.

Pulmonary hypertension (PH) is a prognostically important complication of HFpEF. Progression from isolated post-capillary pulmonary hypertension (IpcPH) to combined post- and pre-capillary pulmonary hypertension (CpcPH) is associated with worse functional capacity and adverse clinical outcomes (Gerges et al., 2024, Assad et al., 2016, Vanderpool et al., 2018). Prior studies have demonstrated higher pulmonary pressures and increased pulmonary vascular resistance among obese HFpEF patients, independent of left-sided filling pressures (Obokata et al., 2017, Reddy et al., 2019). Our data demonstrates an association between obesity and more advanced PH subgroups, suggesting that obesity may contribute to pulmonary vascular remodeling. The biological mechanisms underlying this association remain incompletely defined. Oxylipins regulate vascular tone, endothelial function, and inflammation (Morisseau & Hammock, 2005, Keserü et al., 2008, Pokreisz et al., 2006). In obesity, marked alterations in circulating oxylipin profiles may promote endothelial dysfunction and vascular remodeling (Spector & Kim, 2015). In the setting of HFpEF, such obesity related changes in oxylipin signaling may contribute to the transition from isolated post-capillary PH to combined post- and pre-capillary PH.

Our findings should be interpreted as associative rather than mechanistic. The observed relationships between obesity, PH progression and oxylipin profiles may reflect shared upstream cardiometabolic processes rather than a direct causal pathway. Future studies integrating invasive hemodynamics and oxylipin profiling will be required to determine whether specific oxylipin signatures predict PH progression.

### Limitations

We acknowledge several limitations of our study. First, the relatively small sample size of 90 patients limits statistical power. Hence, the present findings should be interpreted as hypothesis generating and require validation in larger cohorts. Additionally, our binary categorization of obesity (BMI ≥ 30 kg/m²) to preserve statistical power precluded the analysis of overweight patients (BMI 25–29.9 kg/m²) as a distinct intermediate phenotype. Future studies with larger sample sizes are needed to evaluate the step wise impact of adiposity on oxylipin signaling. Second, to mitigate the risk of overfitting because of the limited sample size, we only included age, sex, race, and diabetes as the covariates for our multivariable model. Future studies with larger sample sizes will be able to incorporate a broader range of covariates. Third, although all samples were promptly processed, flash-frozen and stored at − 80 °C to preserve molecular integrity, prolonged storage may introduce variability in the measurement of less stable oxylipin species. This might potentially attenuate observed associations. Fourth, our study cohort was recruited between 2011 and 2012, before the routine use of modern HFpEF therapies, most notably SGLT-2 inhibitors. Because SGLT-2 inhibitors significantly influence metabolism and lipid utilization, their absence in our cohort is a significant limitation. Our data provides important insights into the baseline oxylipin profiles of obesity-related HFpEF, but it is unknown how these oxylipins are modified by modern therapies. Therefore, these findings must be confirmed in newer HFpEF cohorts treated with current guideline directed medical therapy. Fifth, our dataset did not include a non-HFpEF control group or a comparator cohort with HFrEF, as all participants were prospectively enrolled with clinically established HFpEF. Consequently, we are unable to determine whether the observed obesity-related oxylipin alterations are specific to the HFpEF phenotype or if they reflect broader systemic changes associated with adiposity across other heart failure types or in obese individuals without heart failure. Sixth, the cross-sectional design of our study limits inference regarding causality between obesity and oxylipin alterations. Furthermore, our hypothesis regarding the pulmonary uptake of diols or localized altered sEH activity remains speculative based on observational sampling alone. Future studies incorporating direct assessments of enzyme activity are required to substantiate this theory. Seventh, our reliance on BMI as the sole metric of obesity limits our ability to account for adipose tissue distribution. Because waist circumference and specific measures of visceral, subcutaneous and epicardial fat were not collected in our cohort, we cannot determine whether the observed oxylipin alterations are driven predominantly by generalized adiposity or by specific types of fat deposits. Eighth, given the exploratory nature of this study and our relatively small sample size, we did not apply statistical corrections for multiple testing (such as the False Discovery Rate). Hence, we acknowledge an increased risk of false-positive findings. The associations reported here should be interpreted as hypothesis generating and require validation in larger cohorts.

## Conclusion

In patients with HFpEF, obesity is associated with alterations in oxylipin profiles. Obesity in HFpEF is associated with reduced oxylipin diol levels, higher levels of prostaglandin E1 metabolites and a higher burden of pulmonary hypertension. These findings highlight an important associative link between obesity and altered oxylipin signaling in HFpEF.

## Data Availability

The data supporting the findings of this study are available from the corresponding author upon reasonable request.

## Abbreviations

HFpEF: Heart failure with preserved ejection fraction
BMI: Body mass index
PH: Pulmonary hypertension
RV: Right ventricle / right ventricular
RHC: Right heart catheterization
PCWP: Pulmonary capillary wedge pressure
BNP: B-type natriuretic peptide
PUFA: Polyunsaturated fatty acid
sEH: Soluble epoxide hydrolase
EETs: Epoxyeicosatrienoic acids
OR: Odds ratio
CI: Confidence interval
CpcPH: Combined pre- and post-capillary pulmonary hypertension
